# List of reviewers for *Renal Failure* 2022

**DOI:** 10.1080/0886022X.2022.2170009

**Published:** 2023-02-28

The Editors of *Renal Failure* would like to thank all of our reviewers for their contribution and support during 2022. High-quality peer review is essential to the success of the Journal and we greatly appreciate the dedication of all those who have contributed to this.

Abassi, Zaid

Abdelkader, Ahmed

Abdollahi, Ashkan

Abdul Salim, Sohail

Abrahams, Alferso C.

Abreo, Ken

Achanti, Anand

Adam, Ryan

Agostinho Cabrita, Ana de Lurdes

Ahmadian, Elham

Ahmadpoor, Pedram

Akalin, Aysen

Akbulut, Fatih

Akchurin, Oleh

Akhtar, Bushra

Akioka, Yuko

Al-Hwiesh, Abdulla K.

Al-Rawashdeh, Sami

Alaygut, Demet

Albert, Christian

Alexander, Suceena

Ali, Hatem

Allinovi, Marco

Alshogran, Osama

Altintas, Mehmet M

Amasyali, Akin Soner

Amorim, Fábio Ferreira

Andrade-Oliveira, Vinicius

Angioi, Andrea

Aoun, Mabel

Aoyama, Togo

Araú, Maria Júlia Correia Lima Nepomuceno

Arer, Ilker Murat

Asicioglu, Ebru

Assadiasl, Sara

Assumpcao, Lia

Aucella, Filippo

Avramovski, Petar

Ayar, Yavuz

Aziz, Fahad

Aziz, Tavga Ahmed

Azukaitis, Karolis

Badawi, Mohammed

Bai, Yunlong

Balci, Canan

Bandyopadhyay, Bidhan

Barrera-Chimal, Jonatan

Barril, Guillermina

Bartosova, Maria

Barutcu Atas, Dilek

Bashir, Ahmed Muhammad

Basu, Anita

Bátai, István

Bayrakci, Umut

Beck, Laurence H.

Belcher, Justin M.

Bernard, Hory

Bertsias, George K

Besarab, Anatole

Bevc, Sebastjan

Bhalla, Anshul

Bharati, Joyita

Bhatia, Divya

Bihari Bansal, Shyam

Bilen, Nurhan

Bilgili, Beliz

Bingol Ozakpinar, Ozlem

Bird, Victoria Y.

Blanch, Angel

Blaszczyk, Jan

Borolossy, Radwa El

Bottillo, Irene

Bouma, Hjalmar R

Brandi, Lisbet

Branislava, Ilinčić

Bro, Susanne

Budisavljevic, Milos

Bugajska, Jolanta

Bullen, Alexander L

Bunout, Daniel

C Albright Jr, Robert

Cai, Juan

Cai, Guangyan

Cai, Kedan

Cai, Lu

Cai, Qiaoting

Cai, Zhaolun

Çakır, Murat

Calabrese, Vincenzo

Canetta, Pietro A. A.

Cantekin, Işın

Cao, Jing-Yuan

Cappuccilli, Maria

Capusa, Cristina

Caroli, Anna

Casanova, Alfredo

Castaneda, Jorge

Catapano, Fausta

Catar, Rusan

Caza, Tiffany N.

Cellek, Selim

Cetin, Nihal

Chaari, Anis

Chade, Alejandro R.

Chai, Chengzhi

Chakane, Palesa Motshabi

Chandrababu, Ramesh

Charitaki, Evangelia

Chauhan, Kinsuk

Chávez-Iñiguez, Jonathan S.

Chen, Jia

Chen, Chien-Liang

Chen, Chien-Lung

Chen, Fei-fei

Chen, Jin-Bor

Chen, Jing

Chen, Jun-Feng

Chen, Kehong

Chen, Liangmei

Chen, Lvyi

Chen, Xinxin

Chen, Yuanhan

Chen, Yung-Ming

Cheng, Jidong

Cheng, Ziyun

Cheung, Chi Yuen

Cheungpasitporn, Wisit

Chiang, Heng-Pin

Chiba, Takuto

Chopra, Tushar A.

Chua, Horng Ruey

Ciardullo, Stefano

Cicek, Omer Faruk

Cil, Onur

Colussi, Giacomo

Cooney, Robert N.

Cordero-Pérez, Paula

Corrêa, Hugo Luca

Costa, José Abrão

Cremoni, Marion

Crnogorac, Matija

Cseprekál, Orsolya

Cui, Fangqiang

Cui, Liyan

Culpan, Meftun

Cumhur Cure, Medine

Cüre, Erkan

da Fonseca, Cassiane Dezoti

Dabet, Moh'd Mohanad A. Al

Daimon, Shoichiro

Dantas, Marcio

Daoud, Ahmed

Darwin, Darouich

Dasara, Bhavya

Datta, Sudip

Dattolo, Pietro

de Bragança, Ana Carolina

de Pont, Anne-Cornelie

de Sousa, Marcos Vinicius

Debnath, Subrata

Delsante, Marco

Demirci, Cenk

Demirci, Hakan

Deng, Guo-Min

Diao, Zongli

Dika, Živka

Dixit, Mehul P.

Djaiani, George

Djukanovic, Ljubica

Dogan, Ibrahim

Dogra, PavitraManu

Doke, Tomohito

Dong, Yanjun

Dong, Zhe-yi

Dortbudak, Muhammet Bahaeddin

Dos Santos, Daniela

Dossabhoy, Neville R.

Du, Lei

Du, Xin

Du, Zhi Xiang

Duan, Weigang

Dzekova-Vidimliski, Pavlina

Ebrahim, Zarina

Ece, Aydın

Echavarria, Raquel

Edmonston, Daniel L

Efe, Orhan

Eidi, Maryam

Eirini, Grapsa

Ekart, Robert

Ekramzadeh, Maryam

El Borolossy, Radwa

El Rabey, Haddad A

El-Gamasy, Mohamed Abdelaziz

Elbarbary, Ahmed Hussieny

Elmowafy, Ahmed Yahia

Elsaed, Wael

Elshazly, Shimaa M.

Elsheemy, Mohammed

Eren, Hilal

Ergün, Sercan

Eriguchi, Masahiro

Erken, Ertugrul

Ersoy, Fettah

Eşkazan, A. Emre

F Keane, David

Fabbian, Fabio

Faivre, Anna

Farag, Youssef

Fayed, Ahmed

Feng, Ye

Finderup, Jeanette

Fiorentino, Marco

Fitzgibbon, Wayne

Florens, Nans

Fogacci, Federica

Fontana, Francesco

Forbes, Rachel C

Formanowicz, Dorota

Forneris, Giacomo

Fu, Chen

Fu, Haiyan

Fu, Xuebin

Fuentes-Calvo, Isabel

Fuhrman, Dana

Fujikura, Tomoyuki

Fülöp, Tibor

Fung, Winston Wing-Shing

Furuland, Hans

Gaballa, Mohamed

Gameiro, Joana

Gan, Gin-Gin

Gang, Sishir

Gao, Xinghua

Gao, Xuxia

Gao, Yan

Gaipov, Abduzhappar

Gauthier, Alexandre

Ge, Ning

Gebeshuber, Christoph A

Gene L, Bidwell 3rd

George, Jacob

Germain, Michael J.

Gheisari, Yousof

Gilbert, Rodney

Gilbertson, David T.

Gireniz Tatar, Bengu

Gjorgjievski, Nikola

Glenn, Dorey A

Glezerman, Ilya

Goggins, William C

Gökçay Bek, Sibel

Gomez, Rafael

Gondaliya, Piyush

Gööz, Monika

Gorayeb-Polacchini, Fernanda Salomao

Gorek Dilektasli, Asli

Granado, Rolando Claure-Del

Groen In 't Woud, Sander

Gu, Leyi

Gu, Xiangchen

Gui, Yuan

Gumrukcuoglu, Hasan

Gunay, Emrah

Gupta, Rajesh

Gupta, Sanjay

Gupta, Supriya

Guzzi, Francesco

Haase, Micahel

Hall, Isaac E.

Hamdan, Zakaria

Hammes, Mary

Hamrahian, S. Mehrdad

Han, Xue

Han, Jun

Han, Maggie

Han, Mei

Hanset, Nicolas

Harris, Agnes

Hasbal, N. Baris

Hasegawa, Midori

Hashem, Khalid

Hassan Khoso, Mir

Hassan, Hicham I Cheikh

He, Fan

He, Qiqi

Heidari-Beni, Motahar

Heleniak, Zbigniew

Heras, Manuel

Heybeli, Cihan

Hirayama, Kouichi

Ho, Dong-Ru

Hodson, Elisabeth M.

Hong, Quan

Hosohata, Keiko

Hosojima, Michihiro

Hsiao, Ching-Chung

Hsu, Caroline M

Hu, Bo

Hu, Liang

Hu, Shuai

Hu, Yunzhao

Hu, Zhangxue

Hu, Zhizhi

Huang, Xianghua

Huang, Gang

Huang, Jing

Huang, Peipei

Huang, Xianzhang

Huang, Zhihua

Hueso, Miguel

Hui, Wen

Huml, Anne M.

Hung, Ming-Jui

Huo, Guang

Huston, Jessica H

Hwang, Jin Ho

Ichibori, Yasuhiro

Iguchi, Naoya

Ikeda, Masato

Ikegaya, Naoki

Ikenoue, Tatsuyoshi

Imaizumi, Takahiro

Inal, Volkan

Inoue, Tsutomu

Io, Hiroaki

Ionescu, Dorin

Iryaningrum, Maria R

Ishida, Akio

Iwafuchi, Yoichi

Izawa, Atsushi

J, Manjunath

Jaikumkao, Krit

Jain, Nishank

Jalandhara, Nishant

Jamadar, Abeda

Jankovic, Aleksandar

Jemcov, Tamara K.

Jeney, Viktória

Jeng, Yachung

Jha, Pranaw Kumar

Jia, Lan

Jiang, Jun

Jiang, X. -Y.

Jiang, Yuteng

Jin, Peng

Jing-Jing, Zhang

Jung, Hee-Yeon

Jungbauer, Carsten

K, Gokulnath

Kadihasanoglu, Mustafa

Kaewput, Wisit

Kahraman, Dogan

Kakaei, Farzad

Kalantari, Shiva

Kalkan, Yildiray

Kamaruzaman, Lydia

Kandir, Sinan

Kang, Kyung Pyo

Kanic, Vojko

Kant, Sam

Kara, Ali

Karaaslan, Pelin

Karan, Mehmet Akif

Karava, Vasiliki

Karimi, Zynab

Karpaga Selvi, Nachimuthu Maithili

Katagiri, Daisuke

Kataoka, Hiroshi

Kataria, Ashish

Kawamoto, Shinya

Kaya Aksoy, Gülşah

Kaya-Sezginer, Ecem

Kaya, Yasemin

Kaynar, Kubra

Kaypakli, Onur

Ke, Ben

Ke, Lu

Kei-Yan Ng, Andrew

Keshvari, Amir

Khalaf, Mohamed

Khokhar, Manoj

Khor, Ban Hock

Khosravi, Masoud

Khoury, Emad

Kilis-Pstrusinska, Katarzyna

Kim, Gheun-Ho

Kim, Myoung Soo

Kittiskulnam, Piyawan

Kizivat, Tomislav

Klinkhammer, Barbara Mara

Kobayashi, Takaaki

Koch, Christian A.

Koch-Weser, Susan

Kökény, Gábor

Kong, Xianglei

Kopitko, Csaba

Kopp, Jeffrey B.

Kovacs, David Agoston

Kratochwill, Klaus

Kremer, Daan

Kretzamann, Nelson

Kritmetapak, Kittrawee

Krumbholz, Andi

Krzesinski, Pawel

Kulkarni, Yogesh A

Kumar, Ashwani

Kumar, Premranjan

Kumral, Zarife Ozdemir

Kuno, Atsushi

Kurasawa, Shimon

Kurosawa, Shin

Kurtul, Alparslan

Kwon, Osun

Kyriazopoulou, Evdoxia

Lanewala, Ali

Lang, Shi

Lau, Wei Ling

Lazaridis, Miltos

Leavesley, David

Legaz, Isabel

Leisman, Daniel E.

Lengvárszky, Zsolt

Letson, Hayley L

Li, Aiping

Li, Aiqing

Li, Chenyu

Li, Dong

LI, Guisen

Li, Huihui

Li, Jianying

Li, Jianzhong

Li, Jun

Li, Kang

Li, Longkai

Li, Lu

Li, Qiuyang

Li, S.

Li, Xiaobo

Li, Xudong

Li, Yong

Li, Zi

Liang, Yulin

Liangos, Orfeas

Liebman, Scott E.

Liern, Miguel

Lin, Gen-Min

Lin, Hao

Lin, Heng

Lin, Mercury

Lin, Pei-Hui

Lin, Xin

Lin, Xinghui

Lin, Yen-chung

Lingaraju, Madhu C.

Liu, Baoli

Liu, Chan-Min

Liu, Chunyong

Liu, Dong

Liu, Dongwei

Liu, Juan

Liu, Ke-Xuan

Liu, Mei

Liu, Qi-feng

Liu, Tongqiang

Liu, Xing

Liu, Xinhui

Liu, Yueming

Liu, Yuyuan

Liu, Zhangsuo

Liu, Zhiwen

Livingston, Man J.

Lo, Zhiwen Joseph

Loren, Pía

Lu, Limin

Lu, Renhua

Lubbad, Loay

Lucijanic, Marko

Lugli, Gianmarco

Luo, Chuxuan

Lutz, Philipp

lv, Yana

Ma, Haoyang

Ma, Xiaofen

Maanaoui, Mehdi

Macario, Fernando

Machado, Alisson Diego

Maduro, Isolda

Maeda, Akinori

Maeder, Micha T.

Maekawa, Hiroshi

Maesato, Kyoko

Mafi, Alireza

Mahajan, Sandeep

Mahapatra, Amit

Majoni, Sandawana William

Malhotra, Kunal

Malhotra, Sheetal

Malyszko, Jolanta

Manfro, Roberto Ceratti

Manika, Katerina

Manrique-Caballero, Carlos L.

Marckmann, Peter

Mariano, Filippo

Markić, Dean

Martin-Cleary, Catalina

Martinez-Mier, Gustavo

Masajtis-Zagajewska, Anna

Masum, Md Abdul

Matías Opazo Ríos, Lucas

Matono, Takashi

Matsuura, Ryo

Mattana, Joseph

McAdams, Meredith

Mehalingam, Vadivelan

Meier, Clemens M.

Melexopoulou, Christina

Melo, Zesergio

Mendonca, Satish

Meng, Juan

Messchendorp, A Lianne

Metwally, Mohamed M M

Metzinger, Laurent

Meyrier, Alain

Miao, Yun

Miao, Lining

Michael, Ashour

Michishita, Ryoma

Mima, Akira

Ministrini, Stefano

Mirioglu, Safak

Mirzakhani, Mohammad

Mise, Naobumi

Misof, Barbara M

Miura, Kenichiro

Mizuno, Masashi

Mochizuki, Katsunori

Mohebbati, Reza

Moktefi, Anissa

Moldovan, Diana

Molina, Pablo

Molnar, Miklos

Moniwa, Norihito

Monzó, José Jesús Broseta

Moosavi, Seyed

Morello, William

Morisada, Naoya

Morito, Naoki

Morizane, Ryuji

Morris, Earl

Mounira, El Euch

Moyses Neto, Miguel

Mubarak, Muhammed

Mukhi, Dhanunjay

Mulier, J. L. G. Haitsma

Muro, Manuel

Murt, Ahmet

Mushahar, Lily

Musial, Kinga

Nagai, Junya

Nagaraju, Shankar Prasad

Nagasu, Hajime

Nagy, Eman

Nair, Vinay

Naito, Shokichi

Namazi, Soha

Nar, Rukiye

Nardelli, Luca

Nassar, Mahmoud

Nawata, Aya

Nazzal, Lama

Neto, Ricardo

Neves, Precil

Ng, Jack KC

Nie, Zhiqiang

Nigam, Lovelesh

Nigwekar, Sagar

Ning, Yichun

Nishimoto, Masatoshi

Nlandu, Yannick

Nochaiwong, Surapon

Noone, Damien G.

Noppakun, Kajohnsak

Nusca, Annunziata

O'Riordan, Edmond

Oami, Takehiko

Öberg, Carl M

Oh, Dong-Jin

Ohta, Kazuhide

Oliveira-Souza, Maria

Oliveira, Mariana

Oshima, Megumi

Ossareh, Shahrzad

Ostadmohammadi, Vahidreza

Ostróżka-Cieślik, Aneta

Ots-Rosenberg, Mai

Ou, Santao

Ouyang, Dong-yun

Ozcakar, Zeynep Birsin

P de Oliveira, Erick

Pabla, Navjot Singh

Paiste, Henry

Palma, Lilian Monteiro P

Pan, Hao

Pan, Yangbin

Panagoutsos, Stylianos

Pande, Shrikant

Panma, Yuanita

Papasotiriou, Marios

Paripovic, Dusan

Park, Hayne Cho

Park, Sung Bin

Pasquinelli, Gianandrea

Passadakis, Ploumis

Pattharanitima, Pattharawin

Paula de Oliveira Leite, Ana

Peng, Ai

Peng, Hui

Peng, Linlin

Penk, Jamie

Peppelenbosch, Arnoud G.

Pereira, Luciano

Perna, Annalisa

Pethő, Ákos

Petras, Dimitrios

Petrova, Iliyana

Piani, Federica

Piani, Federica

Pike, Mindy

Piko, Nejc

Pistolesi, Valentina

Pleniceanu, Oren

Poloni, José A T

Pontrelli, Paola

Porazko, Tomasz

Portales-Castillo, Ignacio

Posadas Salas, Maria Aurora

Prabhu, Ravindra

Prakash, Jai

Pressly, Jeffrey

Privratsky, Jamie R

Provenzano, Michele

Pruijm, Menno

Przepiorski, Aneta

Puapatanakul, Pongpratch

Qi, Min-You

Qi, Xingshun

Qiu, Ming-zhu

Quigley, Raymond

Quiroz, Roberto Navarro

Rachoin, Jean-Sebastien

Rafati, Foozieh

Rai, Pradeep

Rai, Vikrant

Rajasekaran, Arun

Ralib, Azrina Md

Ramanan, Mahesh

Ramanathan, Ganesh

Ramanathan, Kumaresan

Ramirez-Sandoval, Juan C

Ramirez, Victoria

Rangel, Erika

Rani Kanduri, Swetha

Ranjbaran, Mina

Rao, Indu Ramachandra

Rao, Panduranga

Rasmussen, Sebastian Roed

Rayner, Hugh

Rech, Tatiana H

Ren, Huiwen

Ren, Qiang

Rezapour, Mohammad

Rianthavorn, Pornpimol

Ribeiro, Heitor

Ribeiro, Heitor S.

Ródenas-Alesina, Eduard

Rodríguez-Espinosa, Diana

Roger, Simon D.

Rohloff, Peter

Rong, Liping

Rosenberger, Christian

Rostoker, Guy

Roy, Neil

Rumyansev, Aleksandr

Rush, David N.

Rutkowski, Joseph M

Sabath, Ernesto

Sadioğlu, Rezzan Eren

Sahu, Bidya Dhar

Saifi, Mohd Aslam

Saito, Takao

Sakai, Ken

Sakai, Ryota

Salenger, Page

Salim, Asmat

Samadipour, Ezat

Samejima, Ken-ichi

Sampaio-Maia, Benedita

Sanchez-Lozada, L. Gabriela

Sánchez-Orozco, Laura V

Santoro, Domenico

Santosh, Sadashiv

Scarpioni, Roberto

Schiffl, Helmut

Seeherunvong, Wacharee

Seifi, Behjat

Seixas, Emerson

Selvi, Onur

Senel, Samet

Serafini, Gianluca

Shamsizadeh, Morteza

Shang, Wenjun

Shapiro, Joseph

Sharaf El Din, Usama

Sharma, Purva

Shaw, Brian I

Sheikhbardsiri, Hojjat

Sheldon, Candice

Shemies, Rasha Samir

Shen, Liming

Shen, Xiujin

Shengyou, Yu

Sheshadri, Anoop

Shima, Yuko

Shima, Hisato

Shimada, Satoshi

Shimoda, Masayuki

Shintaku, Sadanori

Shirazian, Shayan

Shum, Hoi Ping

Si, Yachen

Silva Junior, Geraldo

Singh, Manjinder

Singh, Thakur Uttam

Singla, Rohit

Sja'bani, Mochammad

Slee, Adrian

Smith, Susan E

Snauwaert, Evelien

Snelson, Matthew

Sofue, Tadashi

Sokolov, Dmitry

Soliman, Karim

Song, Lu

Sosa-Barrios, Rosa Haridian

Spina, Stefano

Stanton, R. C.

Stavroulopoulos, Aristeidis

Steinman, Theodore I.

Steinwandel, Ulrich

Stepanova, Natalia

Stompór, Tomasz

Strippoli, Raffaele

Strohmaier, Walter Ludwig

Su, Lianjiu

Su, Ning

Suchal, Kapil

Suemitsu, Kotaro

Sugawara, Yasuhiko

Sugimoto, Keisuke

Sugiyama, Masafumi

Sun, Huili

Sun, Luying

Surapaneni, Aditya

Sürmeli Döven, Serra

Suryantoro, Satriyo Dwi

Suvakov, Sonja

Suzuki, Hitoshi

Swanson, Kurtis J

Szabó, Éva

Szamotuslka, Katarzyna

Szolkiewicz, Marek

Tafulo, Sandra

Tagliari, Ana Paula

Taha, Ali S.

Taheri, Saeed

Taherkhani, Amir

Tan, Ruo Yun

Tan, Ying

Tanaka, Hiroshi

Tanaka, Mototsugu

Tanaka, Tetsuhiro

Tang, Wen

Tang, Xiaopeng

Tang, Zhou-Ping

Tao, Xingjuan

Tao, Zhenhui

Tapolyai, Mihály

Taser, Nesibe

Taskapan, Hulya

Teixeira, J Pedro

Thajudeen, Bijin

Thakkar, Jyotsana

Thibaudin, Damien

Thongprayoon, Charat

Tian, Weiqun

Titko, Tetiana

Tong, Fei

Tonyali, Senol

Toprak, Tuncay

Torisu, Kumiko

Torreggiani, Massimo

Tory, Kalman

Trimarchi, Hernan

Trivioli, Giorgio

Tsai, Ming-Tsun

Tsai, Chang-Youh

Tsai, I-Jung

Tsalouchos, Aris

Tseng, Chin-Chung

Tugcu, Murat

Tujios, Shannan

Tunel, Huseyin Ali

Tuokkola, Jetta

Twombley, Katherine

Uddin, Md Jamal

Udomkarnjananun, Suwasin

Ullah, Asad

Ullah, Asmat

Ullian, Michael E.

Unis, Amina

Uppal, Nupur N.

Valeri, Anthony

Valga, Francisco

Valson, Anna T.

van de Logt, Anne-Els

Vega, Jorge

Veighey, Kristin V.

Vela Parada, Xavier

Velarde, Victoria

Verma, Himanshu

VH, Chong

Viegas, Carla

Vnučák, Matej

Wada, Yukihiro

Wahab, Ahmed Mohammed Abd EL

Wan, Jianxin

Wang, Fei

Wang, Chengshi

Wang, Guyan

Wang, Hai

Wang, Hao

Wang, Hua

Wang, Huai-yu

Wang, Jiawu

Wang, Lihua

Wang, Minmin

Wang, Nan

Wang, Ningning

Wang, Pei

Wang, Xianding

Wang, Xinchen

Wang, Yanhong

Wang, Yuefu

Ward, Leigh C.

Wei, Liang

Weinberg, Joel M

Wen, Lu

Whittier, W. L.

Wieruszewski, Patrick M

Wijewickrama, Eranga S

Wong, Michelle M Y

Wong, Chew Ming

Wong, Jiunn

Wu, Ming

Wu, Ping-Hsun

Wu, Suzhen

Wu, Yong

Xia, Yunfeng

Xiao, Jing

Xiao, Jun

Xiao, Wei

Xie, Guangliang

Xie, Lu

Xie, Peng

Xie, Qionghong

Xing, Guolan

Xu, Daoliang

Xu, Fangshi

Xu, Guangtao

Xu, Katherine

Xu, Tao

Xu, Yan

Xu, Yanfang

Xue, Cheng

Yajima, Aiji

Yamada, Shunsuke

Yamanaga, Shigeyoshi

Yamashita, Michifumi

Yamauchi, Junji

Yan, Jianyun

Yang, Dingwei

Yang, Man

Yang, Min

Yao, Qi

Yaribeygi, Habib

Yatim, Karim

Yayla, Muhammed

Yazawa, Masahiko

Yazdanyar, Ali

Yazici, Raziye

Yazıcı Gencdal, Işıl

Ye, Bo

Ye, Guoxin

Ye, Min

Yen, Chieh-Li

Yi, Fan

Yigitbilek, Furkan

Yildiz, Gürsel

Ying-Yong, Zhao

Yngman-Uhlin, Pia

Yong, Tuck

Yoshida, Tadashi

Yu, Xueqing

Yu, Chen

Yu, Dinglai

Yu, Luis

Yu, Xiaofang

Yu, Xueping

Zacchia, Miriam

Zahid, Salman

Zang, Xiujuan

Zeevi, Adriana

Zeiler, Matthias

Zeng, Ming

Zeng, Rui

Zeng, Ying

Zevallos, Jimena Del Risco

Zha, Yan

Zhang, Chao

Zhang, Jiaqiao

Zhang, Yide

Zhang, Dongshan

Zhang, Bo

Zhang, Changmei

Zhang, Dongliang

Zhang, Guangyuan

Zhang, Hengcheng

Zhang, Hong-Liang

Zhang, Kang

Zhang, Li

Zhang, Peng

Zhang, Ti

Zhang, Ting

Zhang, Wei

Zhang, Weiming

Zhang, Wen

Zhang, Xiaoliang

Zhang, Yimin

Zhang, Zhigang

Zhang, Zhijun

Zhang, Zhongheng

Zhao, Shuan

Zhao, Huiping

Zhao, Ping

Zhao, Shuan

Zhao, Shuangping

Zhao, Tingting

Zhao, Wanhong

Zhao, Yan

Zhe, Li

Zheng, Guoping

Zheng, Huijuan

Zheng, Linfeng

Zhou, Guang-Peng

Zhou, Hua

Zhou, Xu-Jie

Zhu, Jiefu

Zhu, Xuejing

Zian, Zeineb

Zimmerer, Jason M

Zorrilla-Vaca, Andres

Zou, Jianzhou

Zou, Lu-xi

Zsom, Lajos

Zununi Vahed, Sepideh

